# COVID‐19 and dementia: experience from six European countries

**DOI:** 10.1002/gps.5497

**Published:** 2021-02-08

**Authors:** Alistair Burns, Antonio Lobo, Marcel Olde Rikkert, Philippe Robert, Norman Sartorius, Maya Semrau, Gabriela Stoppe

**Affiliations:** ^1^ University of Manchester Manchester UK; ^2^ Instituto de Investigación Sanitaria Aragón (IIS‐Aragón) Universidad Zaragoza CIBERSAM National Institute of Health Carlos III (ISC III) Zaragoza Spain; ^3^ Radboudumc Alzheimer Center Radboud University Nijmegen Medical Centre Nijmegen The Netherlands; ^4^ CoBteK Lab and CHU Nice Memory Center University Cote d’Azur Nice France; ^5^ Association for the Improvement of Mental Health Programmes Geneva Switzerland; ^6^ Global Health and Infection Department Brighton and Sussex Medical School University of Sussex Brighton UK; ^7^ University of Basel and MentAge Consulting—Practice‐Research Basel Basel Switzerland

**Keywords:** COVID‐19, dementia, older people

## Abstract

The effects of coronavirus disease 2019 (COVID‐19) have been well documented across the world with an appreciation that older people and in particular those with dementia have been disproportionately and negatively affected by the pandemic. This is both in terms of their health outcomes (mortality and morbidity), care decisions made by health systems and the longer‐term effects such as neurological damage. The International Dementia Alliance is a group of dementia specialists from six European countries and this paper is a summary of our experience of the effects of COVID‐19 on our populations. Experience from England, France, Germany, the Netherlands, Spain and Switzerland highlight the differential response from health and social care systems and the measures taken to maximise support for older people and those with dementia. The common themes include recognition of the atypical presentation of COVID‐19 in older people (and those with dementia) need to pay particular attention to the care of people with dementia in care homes; the recognition of the toll that isolation can bring on older people and the complexity of the response by health and social services to minimise the negative impact of the pandemic. Potential new ways of working identified during the pandemic could serve as a positive legacy from the crisis.

## 1 INTRODUCTION

The worldwide effects of coronavirus disease 2019 (COVID‐19) have been well documented. Older people, and those with dementia, are among the group with the highest COVID‐related mortality and the social consequences of COVID, such as isolation, are likely to affect them disproportionately. People with dementia suffer from the triple jeopardy in that they are: more vulnerable; often neglected or subject to stigma and negative discrimination and less able to look after themselves. There is some evidence internationally that people with dementia are affected negatively by health decisions in relation to COVID^1^ and the longer‐term effects such as neurological damage may also affect them disproportionately.

This paper highlights some aspects of dementia care across six European countries during the current health and social care crisis, based on the experience of the International Dementia Alliance (IDEAL) group and building on our international overview of dementia care services.^2^ Our group, formed in 2002 (formerly the European Dementia Consensus Network), consists of European specialists from six countries with clinical and research experience in the diagnosis and care of people with dementia, their families and carers.

## 2 ENGLAND

There are an estimated 675,000 people with dementia in England, the majority of whom are over 65 and have comorbid health conditions, making them particularly vulnerable to develop severe symptoms and develop complications of their diseases. They are supported by a similar number of carers, most of whom are older people themselves. A quarter of people in acute hospitals and three quarters of residents of care homes (where there are 410,000 beds) have dementia. Key dates in England include:

- 22 January 2020, the Scientific Advisory Group for Emergencies met with senior members of the government.
- 2 March 2020, there were 39 cases in the United Kingdom but no deaths.
- 23 March 2020, ‘lockdown’ was introduced.
- 29 April 2020, mortality figures now include the number of deaths for people in care homes (up to this time, only hospital deaths were recorded).
- 1 May 2020, the Office for National Statistics suggested that there 22,000 excess deaths in care home residents over the previous 4 months.
- 5 November 2020, a second ‘national lockdown’ with restriction of opening of hospitality industry. Care home visiting rules will be revised.

In England (and of relevance to the whole of the United Kingdom), there have been several areas of concern for people with dementia.

It is recognised that people with dementia had specific needs in terms of their response to COVID‐19.^3^ For example, they are much more likely to develop delirium—up to 50% of people in hospital who have dementia also have delirium, twice the rate of those without dementia. Admission to intensive care units was determined by the Rockwood Frailty Scale and NICE highlighted their guidance for hospital admission.^4^ Experience suggested that the delirium at end of life from which people with COVID suffered was unusual in that deterioration would often take place over hours rather than days. General symptoms of malaise as well as specific complaints such as breathlessness are common.

People with dementia may have difficulty understanding complex instructions for isolation and hand washing routines and may have difficulties in expressing some of the symptoms of the disease because of the communication difficulties.^3^ There is also concern that antipsychotic sedative medication was being given inappropriately to people with dementia who wander and were at risk of either being infected or spreading infection.

There were specific issues about end of life care in dementia recognising that death rates would be high. This was compounded by a ban on visitors to care homes (later relaxed to allow one visitor in) and there was publicity about emotional scenes of relatives of people with dementia kissing residents through windows because they were unable to be with them. At the time of writing (December 2020), a pilot study is underway to give a COVID test to care home visitors with the results back in 30 min, allowing the visit to go ahead if the test is negative. The number of people attending funerals was also restricted.

Advice has been amply published to support people with dementia as to how survive self‐isolation.^5^ Services have been negatively impacted with much of the support in the community now being provided remotely. An army of National Health Service supporters (750,000 volunteered) was mobilised to support people at home including providing food to those who were isolated. The issue of remote working in memory clinics has been discussed and the prospect of being able to interview and assess patients, carry out neuropsychological tests and share diagnoses over the telephone or video conferencing.

Personal experience in the epidemic confirmed that many services continued working, and that people looked at innovative ways of maintaining activity.^5^ Interviewing and supporting people on the telephone was generally successful and many older people and their families fully appreciated the need for remote as opposed to face‐to‐face consultation. Indeed, families themselves did not wish to come to hospital for appointments where that could be avoided.

## 3 FRANCE

There are an estimated 900,000 people with dementia in France, the majority of whom are over age 65. In people over 75, two in three residents of institutions have cognitive disorders. 57% of nursing home resident and 70% of long‐term geriatric hospital unit residents suffer from moderate to severe cognitive impairment.

- The French health authorities from the start of the confinement period (which ran from 17 March 2020 to 11 May 2020) and, in particular, why family gatherings were prohibited.
- On 29 May 2020, the statistics indicate^6^ that 28,714 people had died of COVID; 19% and 49% of whom were in nursing homes and in medico social establishments (MSEs).
- Specific monitoring for COVID‐19 has been put in place in nursing home and other types of MSE.

The French Society of Geriatric and Gerontology^7^ underlines that these institutions have difficulties in changing practice because the lack of nursing staff increased the risk of cross transmission.

In parallel to the national statistics and to the global perspective it is also interesting to note that the situations can be very different locally. In fact, this period was an opportunity to use existing resources in a different way, to stimulate fraternity and inventiveness. The following examples come from health facilities in the Southern region but are representative of what has also been achieved in other French regions. This has led to the use of strategies and technologies that have just emerged from research by adapting them to promote the quality of life of patients and families.

The Alzheimer nursing home of the Claude Pompidou Institute in Nice experienced no deaths during this period. The management had, from the beginning of the crisis, asked the nursing staff to no longer work for another establishment by offering financial bonus and suitable conditions for their travel and the care of their children. Psychological aspects highlighted by the crisis should not be overlooked on the patient side of course but also on the family side. From 11 May 2020, the nursing home reopened to the public and family can again but in a supervised manner and with strict barrier measures visit their parents. Before this date, the situation of not being able to meet was a stress factor. The families were both in the situation of losing a loved one and seeking information on the health of that loved one in a place considered to be at high risk. This lead to the promotion of intervention programs targeting the stress response that can be delivered in accessible way. New technologies may have contributed to making this period more acceptable.

There has been a proliferation on the websites of counselling or proposing therapeutic interventions. There was of course the use of telephone calls, video calls allowing an individual relationship. There was also the use of video conference services dedicated to a specific nursing home for families which could both obtain personalised information on their loved one but also share their experience with other families. Finally, certain technological tools usually used for the assessment were modified for interventions in order to provide ‘tailor‐made’ approach and activities depending on individual interests and capacities. During this period clinical practice have informed and modulated the theoretical frameworks. That could be useful for the future.

## 4 GERMANY

Germany has about 1.7 million people with dementia, the majority of whom are cared for by their families and often by relatives who are themselves elderly. Seventy percent of the residents in care homes have dementia.

- COVID‐19 reached Germany at the beginning of March 2020, when the carnival activities were already over.
- In mid‐March, the Federal government decided to impose a lockdown and the recommendations for regulating distance and hygiene were similar to those in other countries.

The older population were regarded as an at‐risk group considered worthy of protection but no separate regulations for individual groups were developed and none of the recommendations stopped people of all ages moving around in public.

The registration of infected and deceased persons is carried out centrally via the local health authorities by the Berlin‐based Robert Koch Institute The Institute publishes daily reports and created a dashboard on which the latest changes can be read.^8^ Despite a successful increase in testing capacity, the recommendations remained that only symptomatic patients should be tested.

Overall, the course of the pandemic has been well managed. so far. By November 2020, over 900,000 corona infections were confirmed. Of these, about 14,000 people have died (4.7 %). Fears that the healthcare system might be overburdened did not materialise. With over 30,000 intensive care places with ventilation in Germany, only a fraction was needed for corona patients. By end of November 2020, a total of 28,800 patients had completed intensive care treatment of whom 23 % died. The mortality rate was particularly high in the over‐70 age group. These people made up 86 % of the deceased, the median being 82 years, that is the age of average life expectancy. Since May 2020, there has been a stable trend of decrease of mortality among people over 80 years of age.

In its statistics, the Robert Koch Institute included nursing homes together with institutions for asylum seekers and prisons. There were no separate statistics for nursing homes for the elderly. At the end of November 2020, 4700 people had died in these institutions, which is more than a third of all those who died in the context of COVID‐19 in Germany. It was also recorded that the people working in these institutions were particularly affected with 49 deaths.

The restrictions on visits to nursing homes were regulated differently from one federal state to another and mostly were also made dependent on the decisions of the owners and managers of nursing homes. These often avoided the slightest risk of infection and were very reluctant to implement relaxation regulations. As a result, residents of care facilities or even dementia residential groups were no longer allowed to leave the house or even their rooms. In some cases, they were also only allowed to eat in their rooms. Access by relatives was either not allowed, or allowed only to a very limited extent. The family members were made aware of the electronic possibilities, for example video telephony. This caused displeasure because all infections that occurred in nursing homes were demonstrably brought in by the staff. The repeatedly demanded regular (serial) testing of nursing home staff has not been implemented to date. For fear of infection, not only the dementia patients but also other residents of the facilities were frequently not allowed outside.

The German Alzheimer's Society produced an information leaflet, in which it advised relatives to draw up an emergency plan in case they fall ill themselves. They were also advised to make use of help of neighbours and of delivery services. Group meetings did not take place, of course, and it is unclear when they will take place again in large numbers. In fact, many people with dementia ultimately died alone because relatives were not allowed to visit them. Meanwhile this situation finds public resonance, for example in the main media. There is an ethical debate, that no one should die alone.

## 5 THE NETHERLANDS

In the Netherlands there are an estimated 250,000 people with dementia, 80,000 of whom are living in nursing homes, and 12,000 of whom are younger than 65 years.

The most relevant timelines for the pandemic spread are:

- 21–25 February: carnival at which there already were severe acute respiratory syndrome coronavirus 2 (SARS‐CoV‐2) carriers who came from Wuhan and Skiing in Italy.
- On 27 February 2020, the first COVID‐19 patient was diagnosed in the Southern part of the country.
- 11 March, over 500 COVID patients
- 15 March, lock down, which meant: closing pubs and schools, citizens could still come out of their houses, but worked at home, general stop of visit possibilities in nursing homes;
- 1 June: end of lock down.

The origin of the extremely high incidence of COVID‐19 among older people in the South were the intimate contacts during the carnival festivities, which still are a famous cultural happening, causing almost all working life to be closed for three days.

During the first month of the Dutch part of the pandemic nearly all public and policy attention was directed at distribution of face masks, COVID‐19 testing tools, and health care professionals to the hospitals. Older persons and professionals in the community and nursing homes were the last in the waiting line. This up till now resulted in almost 10,000 older nursing home patients infected by SARS‐CoV‐2 of whom around a third died.^9^

From 15 March 2020 the nursing homes and other facilities for long term care had a complete lock down, meaning that all older persons were separated from their families. Nursing home professionals had dramatic choices to make: support their older inhabitants without sufficient personal protection, or deliver insufficient personal care and support to them. Moreover, testing was only possible when older patients had two or three of the critical signs (fever, cough or dyspnoea). However, it gradually became clear that the clinical characteristics and outcomes between older and younger patients, and among older patients with a SAS‐CoV‐2 infection are very different. Based on a clinical case and a cohort of 19 patients admitted at a geriatric ward, we highlighted the atypical presentations of this infectious disease, of which severity is linked to frailty, multimorbidity and dementia.^10^ The severity of frailty, multimorbidity and resilience caused COVID‐19 to start often with acute geriatric syndromes such as falls, delirium and dehydration in these patients.

People with dementia, living at home, suffered probably most by the fact that many professional services stopped. Even meal services could not be guaranteed in the midst of our Spring epidemic. The fact that in nursing home patients with or without dementia, the clinical presentation of COVID‐19 and other infections (urinary tract, pulmonary or gastrointestinal infections) could not be separated based on the clinical presentation, made the delivery of adequate care even more difficult. Many became seriously ill and died with shortness of breath and delirium. In palliative care for these patients often just low doses of morphine or benzodiazepines were already alleviating the symptoms sufficiently. Many persons with COVID‐19 and dementia showed an impressive hyper motoric delirium or increase of their challenging behaviour. Remarkably, in our experience some patients with dementia and challenging behaviour also calmed down due to their COVID‐19 infection, probably because their energy level declined.

The lockdown and social isolation of the majority of persons with dementia in the Netherlands during a period of 3 months, is an invasive intervention that had not happened before. Remarkably, there is no clear evidence about the effectiveness of such a lock down as (up to 50% of the) nursing home patients and professionals may still spread SARS‐CoV‐2 in their asymptomatic stage.^9^ Moreover, there are also serious adverse effects of the social isolation and restriction of mobility on immunity, muscle mass, functional performance and last but not least on well‐being and quality of dying.^11^

In summary, the high standards of Dutch dementia care we had before the corona crisis are suddenly seriously under pressure and probably significantly deteriorated. This also led to the chair of the Dutch Alzheimer Society publicly questioning this strict isolation policy in mid‐April.^12^ If we can make the important step of adequate interdisciplinary evaluation and learning of the COVID‐19 crises in dementia care, we may be better prepared for next critical disease periods, and we may even improve proportionality in Dutch dementia care in non‐COVID care times.

## 6 SPAIN

The COVID‐19 pandemic hit hard in Spain. More than seven million individuals in this country are older than 65 years and a quarter of them are octogenarians. It has been estimated that 700,000 individuals aged 40 or more years suffer some kind of dementia.^13^

- The first case of the infection by the COVID‐19 was diagnosed on 31 January 2020 and by 2 June 2020, 239,932 cases have been diagnosed.
- The pandemic has caused officially 40.461 deaths by November, 2020 deaths, although it is generally believed that that the number is much higher. The mortality rate has been high, and particularly high in residential homes.^14^
- The Spanish government issued a Royal Decree to declare a nationwide lockdown starting on 15 March 2020. Following a relaxing, summer period, during the second wave of the epidemic, in November 2020, the responsibility was transferred to the autonomous communities and measures decreed include night limits and curfews.
- An online enquiry in the general population has reported high levels of anxiety, depression and poststress symptoms and older age was among the main associated factors.
- Psychosomatic and liaison psychiatrists have gained an increased recognition in the psychiatric departments because of the care provided for patients in geriatric age admitted to general hospitals.
- Important difficulties for both patients and families, due to the closing of day centres, have been often reported by Alzheimer's disease family associations.

The lockdown has been modified several times, was relaxed during the summer and, at the time of writing this report, in November 2020, during the second wave of the epidemic, the autonomous communities took the responsibility and measures decreed include night limits/curfew in most.

Geriatric psychiatrists immediately alerted the authorities about the potential psychological problems and negative perspectives of the epidemic and the confinement—problems which have been generally confirmed. A large, online enquiry in the general population has reported a high prevalence of anxiety, depression and poststress symptoms (21.6%, 18.7% and 15.8%, respectively), and this morbidity was associated with older age.^15^

Most of the expected difficulties of adaptation of persons with dementia to the confinement have been confirmed. The Spanish Association of Geriatric Psychiatry has explicitly adhered to the position of organisations such as the World Health Organization or Alzheimer's Europe.^16^ The Spanish Psychiatric Association^17^ has also released expert recommendations.

Psychiatrists, like other health professionals working in general hospitals have faced this crisis with an extreme overload of work, often with poor safety conditions. Staff in psychosomatic and liaison psychiatry units, who often take care of patients in geriatric age admitted to general hospitals, have gained an increased recognition from colleagues. During the pandemic they have faced challenges such as the consultations in complex cases of delirium in patients with dementia; the learning and recognition of the adverse effects and interactions of new drugs; the end‐of‐life, psychological support for patients isolated due to the infection or the participation in demanding life and death decisions.

Nursing homes have been severely penalised by the infectious disease and some had a very high infection rate. The mortality rate in these facilities was particularly high when the hospital and intensive care units were crowded and restrictions to hospitalisation in acute wards were implemented. Moreover, the availability of personal protective equipment was considered to be quite deficient. Some residencies run from the family associations have been exceptionally well organised. However, the experience of geriatric psychiatrists is that carers in nursing homes, often with shortness of workforce, have been overburdened during the epidemic. As expected, preventive measures prescribed, such as maintaining social distancing or social isolation was very difficult or impossible. Cases of delirium and severely disturbed behaviour have been commonly observed. It is suspected that concerns about the residents' condition, together with fear of contagion has resulted in an increased level of stress, discomfort and burnout among the professionals in such institutions.

No problems related to provision of food have been reported. However, according to officials in Alzheimer's disease family associations the closing of day centres has resulted in serious difficulties for both patients and families.

Telephone interviewing has also been used successfully for supporting persons with dementia and their families and has been valued by many to substitute for face‐to‐face consultation. In fact, because of the fear of contagion, some families preferred this system rather than going to hospitals for appointments. Technology, home‐based interventions used in some projects before this epidemic by some geriatric psychiatrists is now advocated but was implemented only exceptionally.

## 7 SWITZERLAND

With 8.5 million inhabitants, Switzerland has about 150,000 people with dementia who make up more than 70% of the residents in Switzerland's old people's and nursing homes.^18^ There are many day care and group care services. In Switzerland, the relevant regulations and health care are organised on a cantonal or municipal level.

In the corona pandemic, the Federal government took over the coordination of health care.

- The government reacted swiftly and already at the beginning of March banned the carnival events that were about to begin, in particular the Basel carnival.
- Relaxation steps were taken in several stages, starting on 27 April 2020.
- Beginning in October a second wave arouse. By November 2020, approximately 300,000 infections were detected in Switzerland
- In the Principality of Liechtenstein, 12,000 have been hospitalised, and 3700 have died. By November, almost two and a half million people (with symptoms) had been tested, with 12.3% showing a positive result.

In addition to the cancellation of major events, the general hygiene regulations were advertised, taking into account the linguistic diversity in the country. Just one week later, the Federal Council declared the lockdown in the form that everyone should stay at home. Visits to medical facilities were limited to emergencies. Memory clinics, hospitals, general practitioners' practices, were hardly ever visited. In the old people's homes and nursing homes, a ban on visiting was issued, which applied not only to relatives but also to family doctors and psychiatrists. The decision as to the severity of the implementation was left to the cantons or the institutions and to those managing old people's homes. As a result, it was not possible to monitor the situation in nursing homes from outside for well over a month (with the exception of mails and phone calls). That also means, that no physician and no researcher could monitor clinical symptoms and problems of people with dementia living in nursing homes. In the nursing home itself, protective masks and gloves were not available in the quantity requested during the first weeks. Even if exact figures are missing, in Basel, for example, about half of all COVID‐19 deaths occur in nursing homes. However, in the meantime, many institutions have set up so‐called visitor boxes, which allow contact with relatives using a Plexiglas panel. Contact tracing has been established, and all visitors must wear masks in all institutions and hospitals.

However, the fear of the population went so far that many people, even those with serious illnesses, no longer visited a hospital for fear of becoming infected there. Even in Summer 2020, the medical facilities are still underused. Medical associations and hospitals report more deaths of other conditions. Outpatient services for the elderly, such as day care and group services, have not been available since mid‐March 2020.

Reporting on the infection rate and mortality rate is done centrally by the Federal Office of Health, which prepares a daily report on the situation. With a sharp increase in the frequency of tests, the largest number of reports of new infections had already been received by the end of March 2020. Since then, the rate of new infections and case numbers has been steadily decreasing. Here it became apparent that the older population was particularly affected. The median age of hospitalised persons with corona infections was 71 years, 60% of whom were men.

In Switzerland, the severe encroachment on fundamental rights has led to statements of an ethical nature. At the beginning of May, the National Ethics Committee published ‘ethical considerations in institutions of long‐term care’ in the field of human medicine.^19^ As a rule, the presence of relatives should at least be permitted as a person is dying. The culture of living wills is widespread in Switzerland but there are no statistics on the number of cases affected by COVID infection could die accordingly. In addition, assisted suicide was also prohibited during the lockdown.

The previous telephone visits to the old people's and nursing homes, and the visits throughout the summer and autumn, showed that dementia patients have clearly suffered from the period of isolation. Many of them experienced restlessness and paranoid symptoms, and the psychotropic drug dose often had to be increased. Some of them received psychotropic medications for the first time. The situation for dementia patients at home was worse, because—see above—day clinics and groups have not been offered during this time. However, neighbours helped each other with providing food and intergenerational support developed during this crisis. This was extremely helpful especially for the elderly.

On mid November 2020, finally, the first nursing homes start with regular daily tests of the personal!

## 8 CONCLUSION

Experience across the six European countries represented by the IDEAL group shows, that in spite of individual differences in approach and provision of care, there are common themes in the organisation of services for people with dementia. These include: the early recognition that the presentation of COVID‐19 in older people and those with dementia is often atypical; that people with dementia in care homes often face disadvantages in the provision of care; that isolation of people with dementia and their families can result in a significant toll and; that it is important to take measures to develop the response of services and minimise these effects, in harmony with ethical guiding principles. As COVID‐19 moves from pandemic to endemic, new ways of working will need to be developed to protect the dignity and quality of life for people with dementia and their families.

## CONFLICT OF INTERESTS

Alistair Burns is Assistant Editor of the International Journal of Geriatric Psychiatry.

## Data Availability

The data will be available subject to the usual IP regulations from our host institutions and in line with the journals policy.
